# Effect of distal screw number on axial deformation and fragment-specific stability in AO/OTA 2R3 C2.1 distal radius fractures

**DOI:** 10.1007/s00402-026-06454-6

**Published:** 2026-08-10

**Authors:** Lena Dietz, Christian Spiegel, Felix Christian Kohler, Uta Biedermann, Ivan Zderic, Alicia Feist, Boyko Gueorguiev-Rüegg, Mark Lenz, Wolfram Weschenfelder

**Affiliations:** 1 Department of Trauma, Hand and Reconstructive Surgery and Orthopaedics, Jena University Hospital, Jena, Germany; 2 Institute of Anatomy I, Jena University Hospital, Jena, Germany; 3 AO Research Institute Davos, AO Foundation, Davos, Switzerland

**Keywords:** Distal radius fracture, palmar locking plate, screw number, fracture fixation

## Abstract

**Purpose:**

To investigate the biomechanical influence of distal screw number in palmar plate fixation of unstable intra-articular distal radius fractures with radiodorsal metaphyseal comminution.

**Methods:**

Nine matched pairs of cryopreserved human radii were prepared using a standardized AO/OTA 2R3 C2.1 fracture model with a defined radiodorsal metaphyseal defect zone. For each pair, specimens were randomized to palmar fixation using a 2.4-mm variable-angle locking plate with either four or six distal screws. After embedding, constructs were subjected to cyclic axial loading (150 N, 5,000 cycles) simulating early postoperative rehabilitation. Global axial deformation and fragment-specific three-dimensional interfragmentary motion were recorded using an optical motion-tracking system. Statistical analysis was performed using non-parametric tests (*p* < 0.05).

**Results:**

Two specimens from one donor pair were excluded, leaving 16 radii (8 pairs) for analysis. Bone mineral density and initial construct stiffness did not differ between groups. The six-screw configuration showed significantly lower axial deformation after cyclic loading (*p* = 0.047) and lower radial fragment–shaft translation at both time points (*p* = 0.028; *p* = 0.045). Radial fragment–shaft rotation was lower in the six-screw group at baseline (*p* = 0.018), but no rotational difference remained after cyclic loading. In the overall cohort, axial deformation decreased after cyclic loading (*p* = 0.015).

**Conclusion:**

In this unstable intra-articular fracture model with radiodorsal metaphyseal comminution, six distal screws reduced post-cyclic axial deformation and radial fragment–shaft micromotion compared with four screws. The clinical relevance of these findings remains to be determined.

## Introduction

Palmar locking plate osteosynthesis is the established standard for operative treatment of displaced distal radius fractures [1–4]. Variable-angle systems with multiple distal screw options aim to provide fragment-specific subchondral support, particularly in unstable intra-articular fractures with metaphyseal comminution. Despite their widespread use, implant-related complications and secondary loss of reduction remain relevant concerns, especially in complex patterns and compromised bone quality [5, 6].

It is commonly assumed that increasing the number of distal locking screws enhances construct stability and resistance to fragment displacement. However, available biomechanical evidence is inconsistent. Experimental studies comparing different distal screw numbers have reported no significant differences in construct stiffness or resistance to loss of reduction between four- and higher-screw configurations [7, 8]. Finite element analyses similarly suggest that reducing distal screw number may result in only minor changes in displacement or stiffness, and that screw distribution rather than absolute screw count may be decisive [9, 10]. Conversely, other cadaveric investigations have demonstrated increased axial stiffness with higher distal screw numbers, although without clear evidence of clinically relevant superiority under cyclic loading [11, 12].

Collectively, these findings indicate that the biomechanical relevance of distal screw number remains controversial. Moreover, most studies have focused on extra-articular or simplified fracture patterns and have not specifically addressed unstable intra-articular fractures with defined radiodorsal metaphyseal defect zones—an important contributor to fragment-specific instability [13, 14].

In our previous cadaveric investigations using a standardized AO/OTA 2R3 C2.1 fracture model with radiodorsal comminution, construct augmentation strategies demonstrated fragment-specific effects under cyclic loading [15, 16]. Whether modification of distal screw number alone—within the same palmar plate design—affects global construct behaviour and fragment-specific stability in this unstable intra-articular model has not yet been clarified. The present study forms part of a series of biomechanical investigations using the same validated AO/OTA 2R3 C2.1 fracture model. However, each study addressed a distinct biomechanical hypothesis using independent specimen sets. Previous investigations evaluated construct augmentation using either a headless compression screw or an additional radial plate [15, 16], whereas the present study specifically examines the isolated influence of distal screw number within the same palmar plate system. No specimens or biomechanical data were shared between studies.

Therefore, the aim of the present human cadaveric study was to compare a six-screw distal configuration (three screws per articular fragment) with a four-screw configuration (two screws per fragment) using the same variable-angle palmar locking plate in a standardized AO/OTA 2R3 C2.1 fracture model with a radiodorsal metaphyseal defect zone. The primary outcome parameter was axial construct deformation after cyclic loading. Secondary outcomes included fragment-specific interfragmentary motion and rotational parameters.

## Materials and methods

### Specimens

Nine matched pairs of fresh-frozen human radii (–24 °C) were obtained from the institutional body donation program. Each matched pair consisted of the left and right radius from the same donor, allowing donor-specific matching. Donor age and sex were anonymized by the body donation program and were therefore not available to the investigators. All donors had provided informed consent prior to death for scientific use of their anatomical specimens.

After thawing, the distal 12 cm of each radius was isolated for biomechanical testing. All soft tissues were removed, to standardize specimen preparation and avoid soft-tissue-related variability between specimens. Bone mineral density (BMD, mg/cm³) was measured for all specimens using quantitative computed tomography (qCT; GE Revolution EVO 64, GE Healthcare, Chicago, USA). Volumetric BMD was determined within a metaphyseal region of interest extending proximally from the physeal scar, thereby predominantly assessing metaphyseal cancellous bone relevant to distal screw anchorage.

Two specimens were excluded prior to biomechanical testing due to inadvertent peri-implant fractures that occurred during osteosynthesis preparation before group allocation. Both excluded specimens originated from the same donor pair. Consequently, eight complete matched pairs remained available for analysis, comprising 16 radii with eight specimens in each fixation group. Incomplete optical marker tracking resulted in missing fragment-specific motion data in 2 of 16 measurements for radial-shaft motion, 2 of 16 for ulnar-shaft motion, 1 of 16 for radial-ulnar motion, 1 of 16 for radial-shaft rotation, 1 of 16 for ulnar-shaft rotation, and 4 of 16 for radial-ulnar rotation. Missing values were distributed across both groups and did not affect parameters demonstrating statistically significant between-group differences. The matched-pair design was chosen to reduce inter-specimen variability given the limited availability of human cadaveric specimens.

### Fracture model

A standardized AO/OTA 2R3 C2.1 fracture was created in all specimens to simulate an unstable sagittal intra-articular distal radius fracture with radiodorsal metaphyseal comminution. The sagittal articular osteotomy was placed along the radial ridge between the scaphoid and lunate fossae, corresponding to the level of the third distal plate hole. A radiodorsal metaphyseal defect was then created using two 20° open-wedge osteotomies. The distal extent of the defect was positioned 20 mm proximal to the ulnar articular facet, measured at the ulnopalmar cortical margin, which represented the only preserved metaphyseal cortical contact. Fracture lines were marked on the thawed radii and created using a precision oscillating saw. The same anatomical landmarks, distances, and osteotomy angles were used in all specimens to standardize fracture geometry and defect size across groups.

### Fixation

**Fig. 1:**
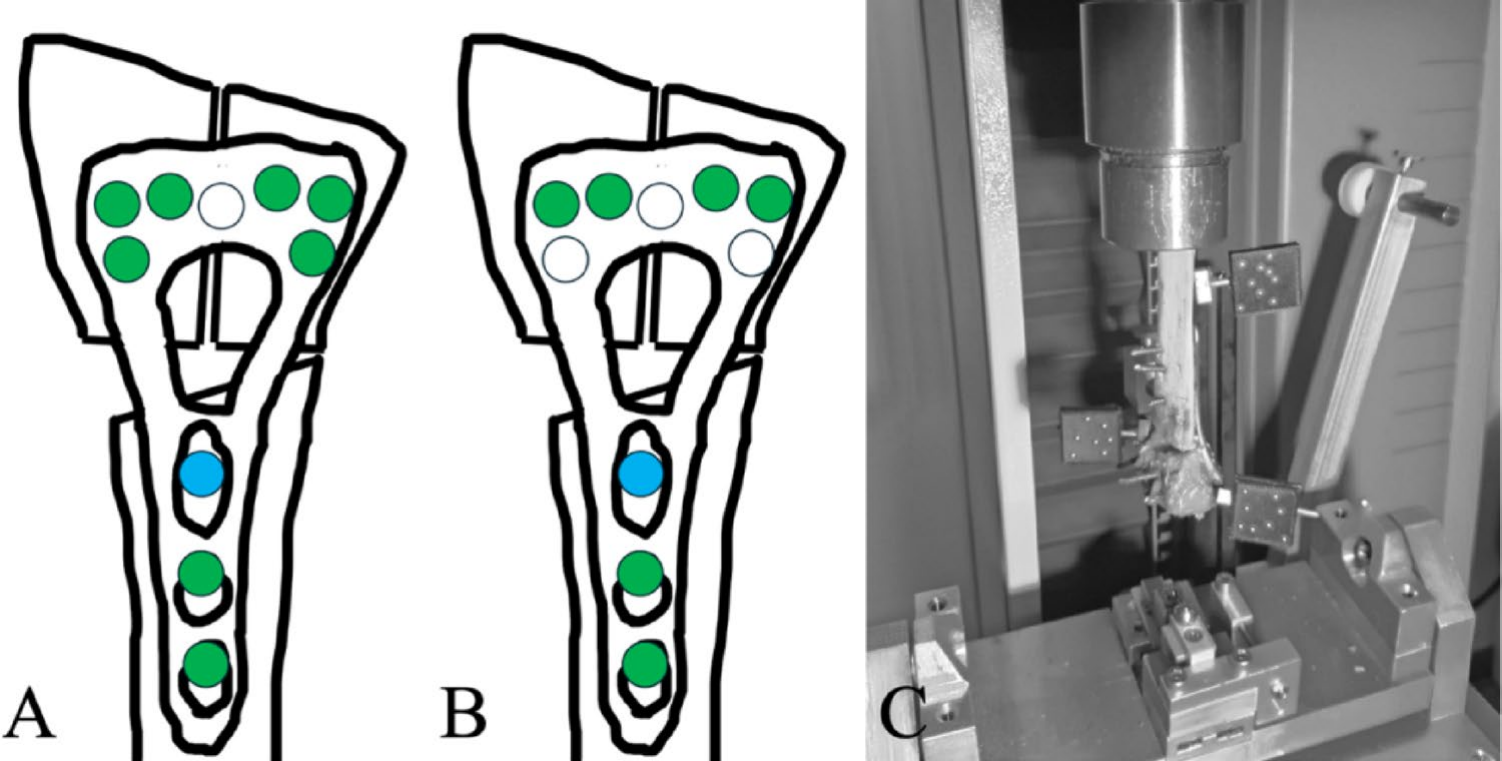
**A** graphical representation of the screw configuration with 6 distal screws (locking screws green, cortical screw blue);** B** graphical representation of the screw configuration with 4 distal screws; C experimental setup with a mounted cadaver radius

Specimens were randomized pairwise to one of two fixation configurations (see Fig. 1):

- 4 distal screws: two distal locking screws per articular fragment placed in the distal subchondral row.
- 6 distal screws: three distal locking screws per articular fragment, consisting of the distal subchondral row plus one additional screw per fragment in the second distal screw row.

All fractures were fixed using the same palmar locking plate system (2.4/2.7-mm VA-LCP distal radius plate 7/3 holes; DePuy Synthes, Zuchwil, Switzerland). The central distal screw hole was intentionally left unoccupied in all specimens. Proximally, the plate was fixed with one cortical and two locking screws in the shaft segment. Screw trajectories and occupied plate holes were standardized for all specimens within each group. Screw insertion torque was standardized to 0.8 Nm using a calibrated torque-limiting screwdriver.

Construct positioning and reduction were visually controlled during preparation, and all osteosyntheses were performed according to a predefined standardized protocol to ensure comparability between specimens. Representative images of the biomechanical test setup are shown in Fig. 1.

### Biomechanical testing

Specimens were proximally embedded in polymethylmethacrylate within custom aluminum fixtures and mounted in a materials testing machine (Zwick 1.0, ZwickRoell, Ulm, Germany). The proximal embedding included the shaft segment proximal to the osteotomy while preserving the distal 12 cm construct for testing. Axial load was transmitted to the distal articular surface via a rocker mechanism with metal spheres, simulating physiological load distribution with approximately 60% of the load applied to the scaphoid fossa and 40% to the lunate fossa. The loading protocol was selected to simulate early postoperative rehabilitation of exercise-stable osteosyntheses and has been widely applied in comparable biomechanical investigations [13–16].

### Testing protocol

- Static baseline measurement at 150 N axial load.
- Cyclic axial loading at 150 N for 5,000 cycles (0.2 Hz).
- Post-cyclic static measurement at 150 N axial load.

Global construct stiffness was calculated from the load–displacement relationship obtained during the static 150 N measurements and was used as a measure of overall construct rigidity. Interfragmentary motion and rotation were recorded using a three-dimensional optical motion-tracking system (Aramis 12 M, Carl Zeiss GOM Metrology GmbH, Braunschweig, Germany). Optical tracking was performed using small rigid 3D-printed marker bodies that were fixed to the shaft and fracture fragments. Each marker body was coated with a stochastic speckle pattern and tracked by the optical measurement system throughout testing. The shaft fragment, radial articular fragment, and ulnar articular fragment were tracked individually throughout testing. Translational motion was defined as the maximum resultant three-dimensional distance change between fragments, whereas rotational displacement was defined as the maximum resultant three-dimensional rotation between fragments. Rotational values therefore represent overall fragment rotation under axial loading and not rotation around a single predefined anatomical axis or isolated torsional resistance of the construct.

### Statistical analysis

Data were analyzed using SPSS (Version 30.0, IBM Corp., Chicago, IL, USA). Normality was assessed with the Shapiro–Wilk test. As data were non-normally distributed, results are reported as median and interquartile range (IQR). Between-group comparisons were performed using the Wilcoxon signed-rank test to account for the matched-pair study design. For fragment-specific outcomes affected by incomplete optical tracking, only donor pairs with valid measurements in both fixation groups were included in the respective between-group comparison. Changes between initial and final measurements within each fixation group were likewise analyzed using the Wilcoxon signed-rank test. The significance level was set at *p* < 0.05.

As this study was designed as an exploratory biomechanical cadaveric investigation with a limited sample size, no formal correction for multiple testing was applied in order to avoid excessive inflation of type II error. Results should therefore be interpreted in an exploratory context.

## Results

Following exclusion of one complete donor pair prior to biomechanical testing, the final study cohort consisted of 16 radii (8 matched pairs), with 8 specimens allocated to each fixation group. Table 1 summarizes BMD and construct stiffness. No significant differences were observed between fixation groups at baseline. In the total cohort, construct stiffness increased significantly from initial to final measurement (*p* = 0.017).

**Table 1:** Bone mineral density and stiffness in palmar plate fixation with 4 distal screws vs. 6 distal screws

Parameter	Group 1 (4 distal screws)	Group 2 (6 distal screws)	*p* between	Entire cohort
Bone Mineral Density (mg/cm³)	167.9 (88.3–182.1)	106.2 (93.8–174.9)	0.78	151.0 (98.1–180.1)
Initial Stiffness (N/mm)	69.7 (63.8–147.9)	123.4 (119.1–194.3)	0.33	115.3 (64.4–170.1)
Final Stiffness (N/mm)	117.2 (85.4–153.0)	146.6 (128.9–182.0)	0.26	128.7 (116.2–173.7)
Change in Stiffness	*p* = 0.098	*p* = 0.156		*p* = 0.017*

Metric values are presented as median with interquartile range (IQR). Between-group comparisons were performed using the Wilcoxon signed-rank test for matched pairs. Within-group comparisons between initial and final measurements were analyzed using the Wilcoxon signed-rank test

* indicating statistical significance with p < 0.05

**Fig. 2:**
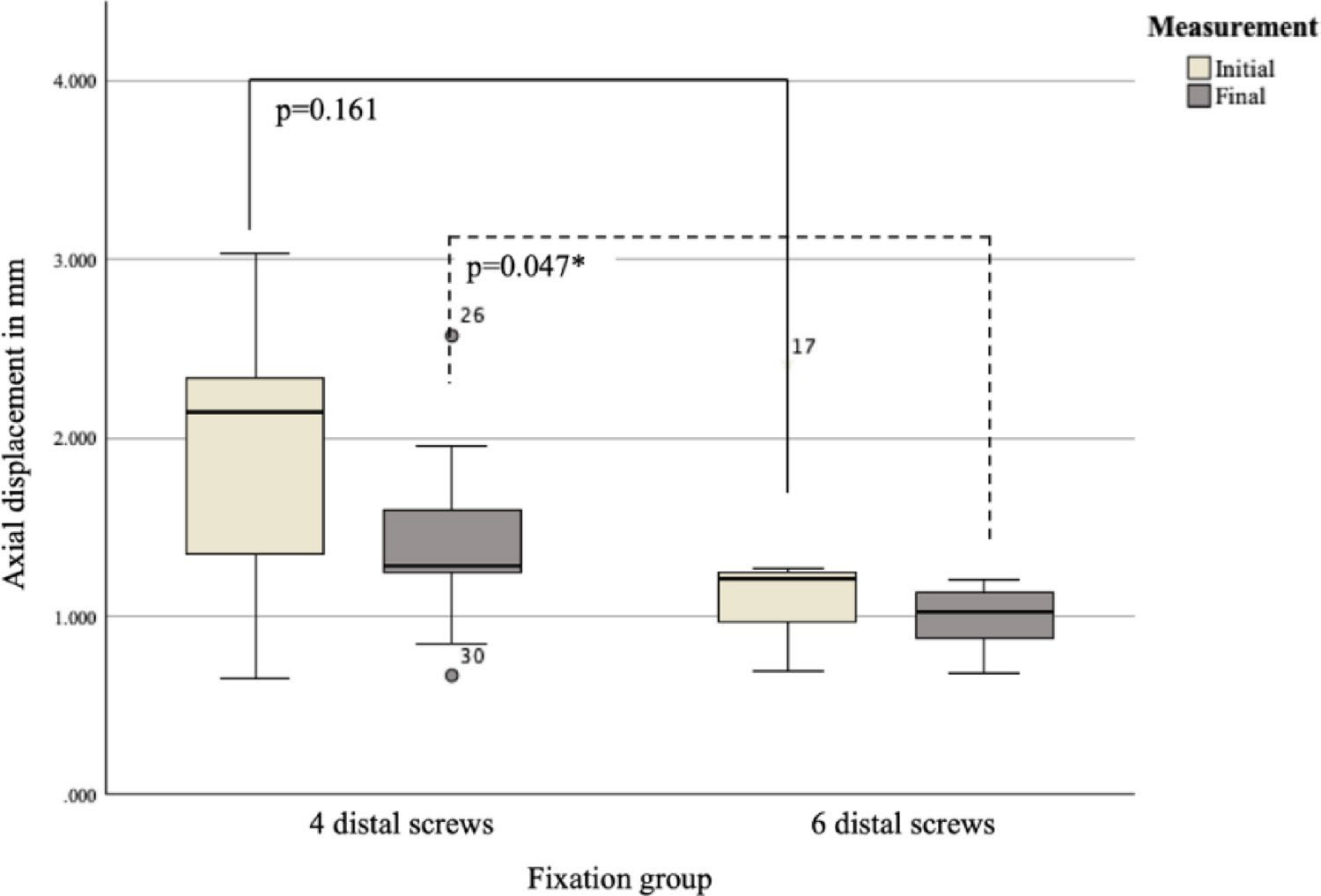
Boxplots of axial deformation at initial and final measurement within both fixation groups

**Fig. 3:**
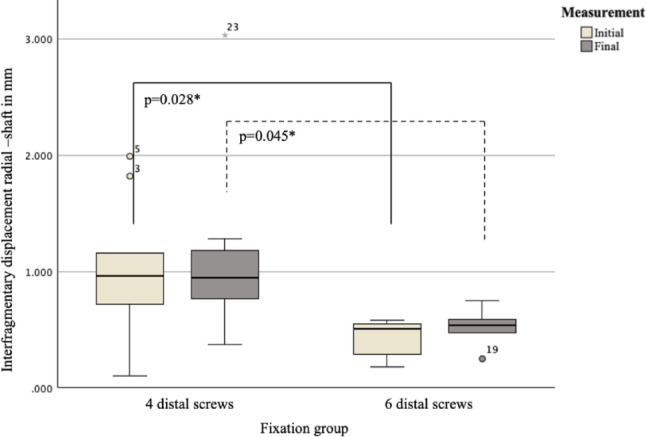
Boxplots of interfragmentary movement of radial articular fragment to shaft fragment at initial and final measurement within both fixation groups

Table 2 presents axial construct deformation and interfragmentary motion measured by optical tracking. Across all translational parameters, lower motion values were observed in the six-screw group. This reached statistical significance for axial deformation at the final measurement (*p* = 0.047, see Fig. 2) and for radial fragment–shaft motion at both time points (*p* = 0.028 and *p* = 0.045, see Fig. 3). In the total cohort, axial deformation decreased significantly between the initial and final measurements (*p* = 0.015).

**Table 2:** Displacement of entire construct and between fragments in palmar plate fixation with 4 distal screws vs. 6 distal screws

ROM Parameter	Measurement	Group 1 (4 distal screws)	Group 2 (6 distal screws)	*p* between	Entire cohort
Axial Displacement (µm)	Initial	2149 (1081–2353)	1216 (774–1263)	0.161	1301 (903–2329)
	Final	1281 (1045–1776)	1025 (824–1167)	0.047*	1189 (865–1291)
	p within	0.098	0.156		0.015*
Radial-Shaft (µm)	Initial	960 (675–1490)	510 (235–565)	0.028*	675 (455–1070)
	Final	950 (738–1233)	535 (415–630)	0.045*	730 (493–1045)
	p within	0.72	0.31		0.26
Ulnar-Shaft (µm)	Initial	785 (553–1915)	435 (398–600)	0.091	565 (448–918)
	Final	940 (580–2380)	490 (320–690)	0.074	630 (435–943)
	p within	0.156	0.59		0.108
Radial-Ulnar (µm)	Initial	225 (158–430)	130 (70–310)	0.74	210 (130–370)
	Final	230 (140–315)	140 (95–275)	0.66	185 (110–293)
	p within	1.00	0.50		0.45

Metric values are presented as median with interquartile range (IQR). Between-group comparisons were performed using the Wilcoxon signed-rank test for matched pairs. Within-group comparisons between initial and final measurements were analyzed using the Wilcoxon signed-rank test

* indicating statistical significance with p < 0.05

Table 3 summarizes interfragmentary rotation. At the initial measurement, radial fragment–shaft rotation was significantly lower in the six-screw group than in the four-screw group (*p* = 0.018). No other significant between-group differences in rotational parameters were observed. Radial fragment–shaft rotation increased significantly between the initial and final measurements in the six-screw group (*p* = 0.031). In the overall cohort, ulnar fragment–shaft rotation increased significantly over time (*p* = 0.033).

**Table 3:** Rotation between fragments in palmar plate fixation with 4 distal screws vs. 6 distal screws

ROT Parameter	Measurement	Group 1 (4 distal screws)	Group 2 (6 distal screws)	*p* between	Entire cohort
Radial-Shaft (°)	Initial	1.94 (1.34–3.03)	1.35 (0.88–1.49)	0.018*	1.41 (1.28–2.48)
	Final	2.13 (1.64–4.30)	1.51 (1.39–2.08)	0.310	1.78 (1.40–2.41)
	p within	0.64	0.031*		0.084
Ulnar-Shaft (°)	Initial	1.84 (1.03–2.94)	1.34 (1.01–1.50)	0.091	1.35 (1.01–2.14)
	Final	1.68 (0.91–2.76)	1.80 (0.72–1.95)	0.92	1.77 (0.86–2.18)
	p within	0.094	0.30		0.033*
Radial-Ulnar (°)	Initial	0.67 (0.33–1.28)	0.66 (0.44–1.14)	0.47	0.67 (0.40–1.23)
	Final	0.59 (0.43–0.74)	0.60 (0.22–0.68)	0.59	0.60 (0.31–0.71)
	p within	0.88	0.125		0.26

Metric values are presented as median with interquartile range (IQR). Between-group comparisons were performed using the Wilcoxon signed-rank test for matched pairs. Within-group comparisons between initial and final measurements were analyzed using the Wilcoxon signed-rank test

* indicating statistical significance with p < 0.05

## Discussion

The present cadaveric study investigated the influence of distal screw number in palmar plate fixation of unstable AO/OTA 2R3 C2.1 distal radius fractures with a defined radiodorsal metaphyseal defect zone. The main findings were that six distal screws resulted in significantly reduced axial deformation after cyclic loading and lower translational micromotion of the radial articular fragment at both measurement time points compared with four screws. Radial fragment–shaft rotation was also lower in the six-screw group at baseline; however, this difference was no longer present after cyclic loading.

The biomechanical relevance of distal screw number remains debated. Several experimental investigations reported no significant differences between reduced and extended distal screw configurations. Moss et al. found comparable stiffness and failure loads in four- versus seven-screw constructs in a cadaveric C2 model [8]. Similarly, Drobetz et al. did not observe substantial improvement in resistance to loss of reduction with additional distal screw rows under cyclic loading conditions [7]. Finite element analyses further suggested that screw distribution rather than absolute screw number may be decisive, with only minor changes in construct behaviour following screw reduction [9, 10].

Conversely, other cadaveric studies demonstrated increased axial stiffness with higher distal screw numbers, although constructs with a reduced number of distal screws were still considered mechanically sufficient in extra-articular fractures [11, 12]. Importantly, most of these investigations were performed in simplified or extra-articular fracture models.

A key difference between the present investigation and several previous biomechanical studies is the fracture model employed. While earlier studies frequently examined extra-articular fractures or fracture configurations with preserved metaphyseal support, the current model incorporated a defined radiodorsal metaphyseal defect zone. As a consequence, load transfer was largely dependent on the palmar-ulnar cortical buttress and the implant construct itself, with only limited residual metaphyseal support. This configuration may more closely resemble osteoporotic distal radius fractures with metaphyseal comminution encountered in clinical practice.

Under these conditions, additional distal screws may provide increased subchondral support and improve support of the radial articular fragment and load transfer to the shaft. This may explain why a significant reduction in axial deformation and radial fragment micromotion was observed in the present study, whereas previous investigations reported little or no biomechanical advantage of increasing distal screw number. Our findings therefore suggest that screw number may be particularly relevant in fracture patterns characterized by asymmetric metaphyseal support and compromised radial column stability.

Although initial construct stiffness was numerically lower in the four-screw group than in the six-screw group, this difference did not reach statistical significance. Given the small sample size, however, limited statistical power rather than true equivalence cannot be excluded. As the six-screw group also demonstrated lower axial deformation after cyclic loading, a contribution of baseline construct differences to the observed group effect cannot be ruled out and should be considered when interpreting the results.

The significant increase in construct stiffness and the concomitant decrease in axial deformation after cyclic loading are consistent with an initial settling or bedding-in effect of the specimen–fixture and implant–bone construct. Improved seating of the articular loading interface and fixation components during the initial loading cycles may explain these changes more plausibly than true structural strengthening of the osteosynthesis. Accordingly, changes over time should primarily be interpreted as a characteristic of the experimental setup.

A significant between-group difference in radial fragment–shaft rotation was observed at baseline but not after cyclic loading. Because the difference was already present before cyclic testing and did not persist at the final measurement, it does not provide evidence of improved rotational stability through the use of additional distal screws. Radial fragment–shaft rotation nevertheless increased significantly over time within the six-screw group. However, the absolute median change was small (< 0.2°), and no corresponding final between-group difference was detected. Given the limited sample size and exploratory nature of the study, the biomechanical relevance of these findings remains uncertain.

From a clinical perspective, reducing distal screw number has been proposed to lower implant-related costs and potentially reduce implant-related complications [8–10, 12]. The present findings suggest that while four distal screws provided measurable stability, six screws were associated with lower axial deformation after cyclic loading and reduced translational motion of the radial articular fragment in unstable intra-articular fractures with radiodorsal metaphyseal comminution. Nevertheless, the clinical relevance of these biomechanical differences remains uncertain. Although six distal screws reduced motion under controlled laboratory conditions, it cannot be concluded that these differences necessarily translate into reduced secondary displacement, lower complication rates, or improved functional outcomes in patients. Maintenance of fragment position is generally considered desirable in unstable intra-articular fractures, particularly in the presence of metaphyseal comminution and limited cortical support. Future clinical studies are required to determine whether the biomechanical advantages observed in this experimental model are associated with meaningful patient-related benefits.

### Strengths and limitations

A matched-pair human cadaveric design and a standardized unstable intra-articular AO/OTA 2R3 C2.1 fracture model with a defined radiodorsal metaphyseal defect zone allowed controlled intergroup comparison. Fragment-specific three-dimensional motion tracking enabled detailed assessment beyond global construct stiffness.

Limitations include the relatively small sample size, the exploratory nature of the statistical analysis without adjustment for multiple comparisons, and the inherent constraints of cadaveric testing without biological healing. Although axial compression represents a widely accepted loading scenario for biomechanical testing of distal radius osteosyntheses and simulates early postoperative rehabilitation, only axial loading was investigated. Consequently, the present findings cannot be extrapolated to multidirectional loading conditions encountered during daily activities or accidental support reactions.

Furthermore, only one commercially available variable-angle palmar locking plate system was evaluated. Because plate geometry, distal screw orientation, and available screw trajectories differ between implant systems, the present findings should be regarded as specific to the tested construct and cannot necessarily be generalized to other implant designs.

Interfragmentary motion was assessed under standardized static loading conditions before and after cyclic loading. Continuous peak motion during the 5,000 loading cycles was not recorded and therefore transient maximum fragment displacement during cyclic loading cannot be assessed. Incomplete optical marker tracking resulted in missing values for selected fragment-specific parameters. Missing values were not imputed. Between-group analyses of affected parameters were restricted to donor pairs with valid measurements in both fixation groups, thereby reducing the effective sample size for selected comparisons.

Finally, donor age and sex were anonymized by the body donation program and were therefore unavailable to the investigators. Although the standardized fracture model represents an osteoporotic injury pattern with radiodorsal metaphyseal comminution, direct extrapolation of the present findings to specific patient populations should be made with caution.

## Conclusion

In conclusion, increasing distal screw number from four to six in palmar plate fixation of unstable AO/OTA 2R3 C2.1 fractures with radiodorsal metaphyseal comminution reduced axial deformation after cyclic loading and radial fragment micromotion. The clinical relevance of these biomechanical differences remains to be determined.

## Data Availability

The data presented in this study are not publicly available but available on request from the corresponding author. The data are not publicly available due to privacy and ethical restrictions.

## Abbreviations

AO: Arbeitsgemeinschaft für Osteosynthesefragen (Association for the Study of Internal Fixation)
BMD: Bone mineral density
CT: Computed tomography
IQR: Inter quartile range
LCP: Locking compression plate
qCT: Quantitative computed tomography
VA-LCP: Variable angle locking compression plate
